# Estrogen protects SGC7901 cells from endoplasmic reticulum stress-induced apoptosis by the Akt pathway

**DOI:** 10.3892/ol.2013.1701

**Published:** 2013-11-22

**Authors:** ZHENGQI FU, FENG ZOU, HAO DENG, HONGYAN ZHOU, LIJIANG LIU

**Affiliations:** 1 Department of Pathology and Pathophysiology, School of Medicine, Wuhan, Hubei 430056, P.R. China; 2 Jiangda Pathology Institute, Jianghan University, Wuhan, Hubei 430056, P.R. China

**Keywords:** endoplasmic reticulum stress, Akt, gastric cancer, apoptosis

## Abstract

Several previous studies have demonstrated that estrogen may protect cancer cells from endoplasmic reticulum stress-induced apoptosis. However, the molecular mechanisms involved are not fully understood. In the present study, human gastric adenocarcinoma SGC7901 cells were treated with tunicamycin (TM) to induce endoplasmic reticulum stress. This was demonstrated by increased glucose-regulated protein 78 expression and enhanced phosphorylation of protein kinase RNA-like endoplasmic reticulum kinase. Endoplasmic reticulum stress induced caspase-3-mediated apoptosis with the inhibition of Akt; the latter of which was measured by the activity-dependent phosphorylation at Ser473 of Akt. Simultaneous treatment of 10^−9^ M 17β-estradiol (E2) with TM may protect SGC7901 cells from endoplasmic reticulum stress-induced apoptosis by counteracting the inhibitory effect of TM on Akt, causing an increase in the phosphorylation of Ser473-Akt. It was concluded that low concentrations of E2 may counteract endoplasmic reticulum stress-induced inactivation of Akt to block caspase-3-mediated apoptosis.

## Introduction

Gastric cancer is one of the most common malignancies in China and is a leading cause of cancer-related mortality worldwide (1–3). Previous epidemiological studies have shown that the average age of disease onset in females is usually delayed by ~15 years compared with that in males. However, the incidence following menopause in females is close to that in males (4). Hormonal factors associated with a greater exposure to estrogen and/or progesterone may be associated with the decreased risk of gastric cancer (5). From a previous male cohort study of patients with prostate cancer in Sweden, estrogen exposure resulted in a decrease in the risk of gastric cancer (6). These observations suggested that estrogen is pivotal in gastric cancer. However, the mechanism of estrogen signaling in gastric carcinogenesis has not been well established.

The endoplasmic reticulum is an organelle responsible for protein folding and assembly, lipid and sterol biosynthesis and free calcium storage. A number of biochemical, physiological and pathological stimuli, such as those that cause endoplasmic reticulum calcium depletion, altered glycosylation, nutrient deprivation, oxidative stress or hypoxia, lead to endoplasmic reticulum stress, in which numerous rescue responses, including unfolded protein response (UPR), are triggered. To adapt to stress conditions, the concerted action of three endoplasmic reticulum transmembrane proteins, protein kinase RNA-like endoplasmic reticulum kinase (PERK), inositol-requiring enzyme-1 (IRE1) and activating transcription factor (ATF) 6, are activated and protect cells by an initial decrease in general protein synthesis, promotion of protein folding via the induction of chaperones [such as glucose-regulated protein 78 (GRP78)] and prevention of accumulating misfolded proteins. However, if the stress is severe or prolonged, distinct death signals may be transduced during the UPR and cells undergo apoptosis (7,8). Several previous studies have shown that in various tumors, such as gastric cancer, whose cells experience increasing nutrient starvation and hypoxia, endoplasmic reticulum stress is highly induced and closely associated with cancer cell death mediated by ATF4 and C/EBP homologous protein (CHOP) (9,10).

Furthermore, previous studies have found that apoptosis induced by endoplasmic reticulum stress may be affected by the estrogen signaling pathway. Estrogen may induce GRP78, which has been found to correlate with cell viability and resistance to paclitaxel and cisplatin in endometrial cancer (11). Administration of a small volume of 17β-estradiol (E2)prolonged the survival of rats by 3 h by ameliorating endoplasmic reticulum stress (12). Isoflavones, that have a structure similar to that of E2 and are capable of binding to estrogen receptors with seven to eight times less binding affinity to estrogen receptor (ER) α than to ER β, protected SH-SY5Y cells from cell death by suppressing endoplasmic reticulum stress. This was determined by decreased expression of GRP78 mRNA, spliced X-box binding protein-1 mRNAs and CHOP (13). These findings suggested that low concentrations of estrogen may protect cancer cells from apoptosis induced by endoplasmic reticulum stress. In addition, they provide marked explanations for previous epidemiological observations that the incidence of gastric cancer following menopause in females increases and is close to that in males, while estrogen exposure results in a decrease in the risk of gastric cancer. However, the molecular mechanism by which estrogen at low concentrations protects gastric cancer cells from endoplasmic reticulum stress-induced cell death remains unclear.

Therefore, the present study treated SGC7901 cells with tunicamycin (TM), which is well known to induce endoplasmic reticulum stress by inhibiting N-linked protein glycosylation (14,15). Cells were then treated with TM plus E2 at a nanomolar concentration (10^−9^ M). The endoplasmic reticulum stress induced by TM was found to result in apoptosis with the inhibition of Akt. In addition, the simultaneous treatment of E2 with TM may protect SGC7901 cells from endoplasmic reticulum stress-induced apoptosis by the Akt pathway.

## Materials and methods

### Antibodies and chemicals

The 17β-estradiol (E2) was purchased from Sigma-Aldrich (St. Louis, MO, USA), rabbit anti-GRP78 was purchased from Abcam (Cambridge, UK) and pAb against phospho-Akt at Ser473 (Ser473-Akt) was obtained from Cell Signaling Technology, Inc. (Danvers, MA, USA). pPERK (Thr 981) and anti-β-actin (C4) antibodies were purchased from Santa Cruz Biotechnology, Inc. (Santa Cruz, CA, USA). TM was purchased from Alexis Biochemical Corp. (San Diego, CA, USA), dissolved in DMSO at a concentration of 3 mM and stored at −20°C. Bicinchoninic acid protein detection kit, goat anti-mouse peroxidase-conjugated secondary antibody, chemiluminescent substrate kit and polyvinylidene difluoride (PVDF) membranes were purchased from Pierce Biotechnology, Inc. (Rockford, IL, USA). RIPA buffer and enhanced chemiluminescence reagents were purchased from Beyotime Institute of Biotechnology (Haimen, China).

### Cell culture and treatment

The human gastric adenocarcinoma cell line, SGC7901, was obtained from the Cell Center of Basic Medicine, Chinese Academy of Medical Sciences (Beijing, China). Cells were cultured in RPMI-1640 containing 10% fetal calf serum, at 37°C in a 5% CO_2_ atmosphere. To study the effect of TM on endoplasmic reticulum stress, the cells were treated with TM at various concentrations. In addition, to explore the protective potential of E2 in endoplasmic reticulum stress-induced apoptosis, various concentrations of E2 were administered in the TM-treated cells and the same concentrations of DMSO and alcohol were used as vehicle control.

### Cellular viability using WST-1 test

Viability of SGC7901 cells treated with TM and cotreated with TM plus E2 was measured using a WST-1 cell counting kit (Beyotime Institute of Biotechnology) according to the manufacturer’s instructions. SGC7901 cells were seeded in 96-well culture plates in the media for 48 h and treated with various concentrations of TM, with and without various concentrations of E2, for 48 h. Corresponding controls with analogous concentrations of DMSO and alcohol were performed in parallel. The cells were then incubated with WST-1 reagent for 1 h at 37°C. The absorbance at 450 nm was monitored and the reference wavelength was set at 630 nm. The percentage viability of cells was calculated by comparison with that of control cells (16).

### Western blot analysis

Western blot analysis was performed according to the methods previously established (17,18) and cultured cells were directly lysed with a RIPA buffer. The protein concentration was measured using the bicinchoninic acid kit according to the manufacturer’s instructions. Next, proteins were separated by 10% SDS-polyacrylamide gel electrophoresis and transferred to PVDF membranes. The membranes were blocked with 5% non-fat milk dissolved in TBS Tween-20 [50 mM Tris-HCl (pH 7.6), 150 mM NaCl and 0.2% Tween-20] for 1 h and probed with primary antibodies at 4°C overnight. The blots were then incubated with anti-mouse or -rabbit IgG conjugated to horseradish peroxidase (1:5,000) for 1 h at 37°C. The blots were visualized with enhanced chemiluminescence and quantitatively analyzed using the TotalLab analysis software (Nonlinear USA Inc., Durham, NC, USA).

### Statistical analysis

Data are expressed as means ± standard deviation and were analyzed using SPSS 12.0 statistical software (SPSS Inc., Chicago, IL, USA). The one-way analysis of variance procedure followed by the least significant difference post hoc test were used to analyze the differences among groups.

## Results

### TM induces endoplasmic reticulum stress in SGC7901 cells

**Figure 1:**
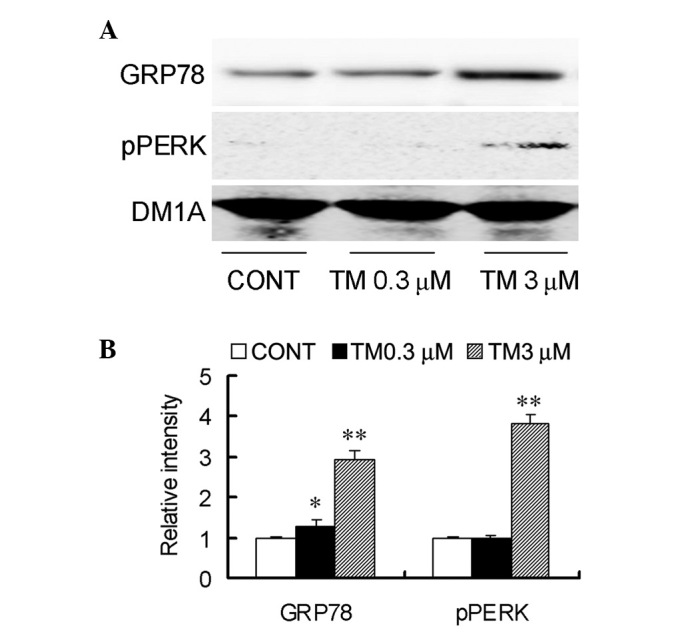
TM-induced endoplasmic reticulum stress in SGC7901 cells. SGC7901 cells were treated with TM at concentrations of 0.3 and 3 μM for 24 h. The same volume of DMSO was injected as a relative control. Levels of relative endoplasmic reticulum stress determined by specific antibodies (GRP78 and pPERK) were (A) measured by western blot analysis and (B) quantitatively analyzed. Data are presented as the means ± SD of three independent experiments. ^*^P<0.05 and ^**^P<0.01, vs. the control. TM, tunicamycin; PERK, protein kinase RNA-like endoplasmic reticulum kinase; GRP78, glucose-regulated protein 78.

To produce an endoplasmic reticulum stress model *in vitro*, SGC7901 cells were treated with TM at concentrations of 0.3 and 3 μM for 24 h. Next, the expression of GRP78 and pPERK, the ER stress markers, were detected. Treatment with the two concentrations of TM were found to increase the protein levels of GRP78, whereas the increased levels of pPERK were only detected at 3 μM (Fig. 1). These results confirmed the *in vitro* induction of endoplasmic reticulum stress by TM.

### Endoplasmic reticulum stress induced by TM results in apoptosis

**Figure 2:**
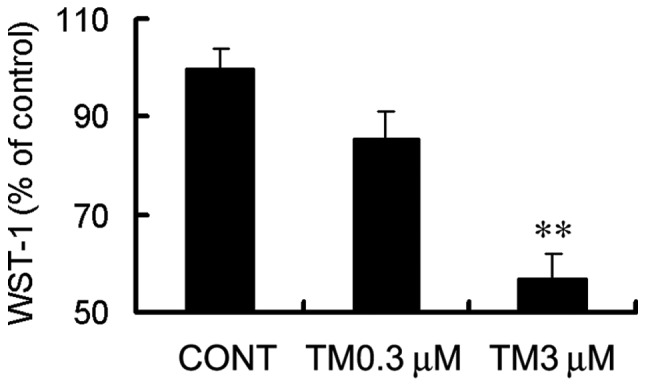
Endoplasmic reticulum stress induced by TM results in cytotoxicity. SGC7901 cells were treated with TM at concentrations of 0.3 and 3 μM for 48 h. The same volume of DMSO was injected as a relative control. Cell viability was evaluated using the WST-1 test. Data are presented as ratios of the control levels and are the means ± SD from three independent experiments. ^*^P<0.05 and ^**^P<0.01, vs. the control. TM, tunicamycin.

**Figure 3:**
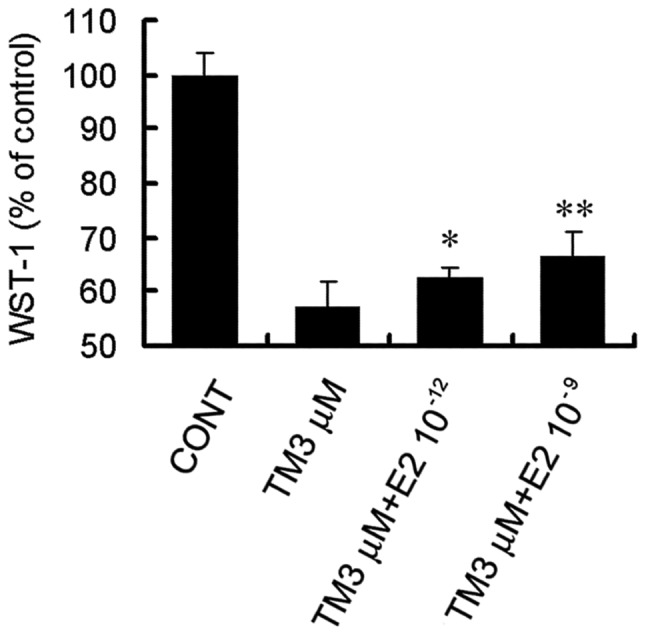
E2 protects SCG7901 cells against cytotoxicity induced by endoplasmic reticulum stress. SGC7901 cells were treated with 3 μM TM with or without E2 at 10^−12^ and 10^−9^ M for 48 h. The same concentrations of DMSO and alcohol were used as control. Cell viability was evaluated using the WST-1 test. Data are presented as ratios of the control levels and are the means ± SD from three independent experiments. ^*^P<0.05 and ^**^P<0.01, vs. the control. TM, tunicamycin; E2, estradiol.

**Figure 4:**
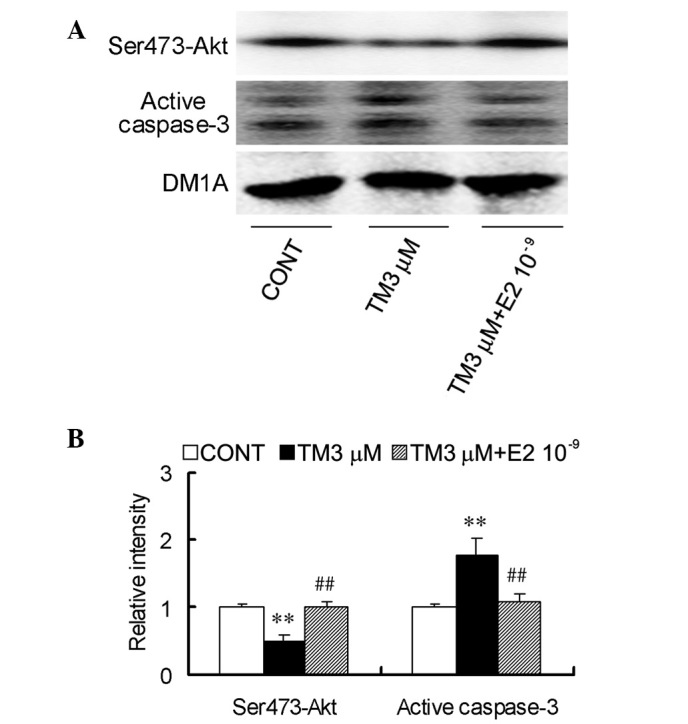
E2 protects SCG7901 cells from endoplasmic reticulum stress-induced apoptosis by the Akt pathway. SGC7901 cells were treated with 3 μM TM with or without E2 at 10^−9^ M for 24 h. The same concentrations of DMSO and alcohol were used as control. The levels of active caspase-3 and the activity status of phospho-Ser473-Akt were measured by (A) western blot analysis and (B) quantitatively analyzed. Data are presented as the means ± SD of three independent experiments. ^*^P<0.05 and ^**^P<0.01, vs. the control. TM, tunicamycin; E2, estradiol.

TM is a nucleoside antibiotic that leads to apoptosis, by inhibiting the N-glycosylation of target asparagine residues in the luminal domains of proteins (19). Using a viability assay (WST-1 test), TM treatment at a concentration of 3 μM was found to result in evident cytotoxicity (Fig. 2) and 3 μM TM treatment for 24 h increased the production of 17- and 19-kDa activated caspase-3 (Fig. 4).

### E2 protects SCG7901 cells against apoptosis induced by endoplasmic reticulum stress

To determine the effect of E2 on endoplasmic reticulum stress-induced cytotoxicity, cells were cotreated with 3 μM TM and various concentrations of E2 for 48 h. E2 significantly attenuated cytotoxicity at the two concentrations of 10^−12^ and 10^−9^ M (Fig. 3). E2 at 10^−9^ M was found to have a significant effect and was selected for further experiments.

To further confirm the protective effect of E2 on endoplasmic reticulum stress-induced apoptosis, protein levels of the cleavage of procaspase-3 to active caspase-3 fragments were measured. The production of 17- and 19-kDa activated caspase-3 was found to decrease following cotreatment with 3 μM TM and 10^−9^ M E2 for 24 h (Fig. 4).

### E2 protects SGC7901 cells from endoplasmic reticulum stress-induced apoptosis by the Akt pathway

Akt, a serine/threonine protein kinase that regulates the balance between cell survival and apoptosis, has been previously reported to be involved in endoplasmic reticulum stress-induced apoptosis. It was tested whether TM affects the activation-associated phosphorylation of Ser473 of Akt and whether E2 protects SGC7901 cells from endoplasmic reticulum stress-induced apoptosis by the Akt pathway. Treatment with 3 μM TM was found to greatly decrease Ser473-Akt immunoreactivity (Fig. 4) and cotreatment with 10^−9^ M E2 counteracted the inhibitory effect of TM on Akt, causing an increase in Ser473-Akt (Fig. 4).

Overall, these results indicated that E2 is able to counteract endoplasmic reticulum stress-induced inactivation of Akt to block signaling to caspase-3.

## Discussion

In the present study, the induction of endoplasmic reticulum stress by treatment with TM was found to induce apoptosis with the inhibition of Akt. Simultaneous treatment of 10^−9^ M E2 with TM was found to arrest endoplasmic reticulum stress-induced apoptosis by counteracting the inhibitory effect of TM on Akt, causing an increase in phospho-Ser473-Akt. It was concluded that low concentrations of E2 are able to counteract endoplasmic reticulum stress-induced apoptosis by Akt pathway.

Endoplasmic reticulum stress and UPR are highly induced in various tumor types, such as gastric cancer, whose cells possess rapid glucose metabolism and fast growth rate, which lead to poor vascularization of tumor mass, low oxygen supply, nutrient deprivation, pH changes and express mutant proteins that do not fold correctly. The primary role of the UPR is to provide survival signaling pathways required for tumor growth by dissociating GRP78, which regulates the protein folding process, from three endoplasmic reticulum stress sensors (including PERK, IRE1α and ATF6) that are consequently phosphorylated and activated. However, if the attempt to recover from endoplasmic reticulum stress fails, UPR induces cell death programs to eliminate the stressed cells (14). It has been previously reported that the PERK-mediated α-subunit of eukaryotic translation initiation factor (eIF2α) phosphorylation, which contributes to the attenuation of translation to alleviate stress damage in the early stage, may selectively initiate the translation of ATF4 mRNA. This subsequently activates the expression of genes involved in endoplasmic reticulum stress-associated apoptosis (9,20). GADD153/CHOP, downstream of the PERK/eIF2α pathway, mediates endoplasmic reticulum stress-induced apoptosis (10). In the current study, endoplasmic reticulum stress induced by TM was shown to result in caspase-3-mediated apoptosis in SGC7901 cells.

Gastric tumor has been generally considered as a non-estrogen related tumor. However, a growing number of previous epidemiological observations have shown that the ratio between the male and female incidence of gastric cancer is between 2:1 and 3:1. This difference disappears in females following menopause, and in addition to estrogen levels, this difference is difficult to explain with any one of the other known risk factors (21). Oral contraceptives and estrogen replacement therapy reduce the incidence of gastric cancer (22). The incidence of gastric cancer has been found to increase in females receiving oophorectomy and to decrease in females receiving estrogen replacement therapy (5). The incidence of gastric cancer in males with prostate cancer administered E2 treatment is considerably lower than that in males who have not received treatment (6). In our previous study, estrogen at high concentrations inhibited the growth of gastric cancer cells, while at low concentrations promoted cell growth (data not published). This highlighted marked explanations to the epidemiological mystery that the incidence of gastric cancer in males has been considerably higher than that in females. In addition, estrogen may induce GRP78, which has been found to correlate with cell viability and resistance to paclitaxel and cisplatin in endometrial cancer (11). Administration of a small volume of E2, prolonged the survival of rats by 3 h by ameliorating endoplasmic reticulum stress (12). Isoflavones, which have a structure similar to that of E2, may protect SH-SY5Y cells from cell death by suppressing endoplasmic reticulum stress. In the current study, 10^−9^ M E2 was found to counteract endoplasmic reticulum stress-induced apoptosis.

Akt, also known as protein kinase B, is a serine/threonine protein kinase that has been shown to regulate the balance between cell survival and apoptosis (23,24). Misregulation of the Akt signaling pathway has been found to play a central role in tumorigenesis. Activation of Akt tips the balance of cells into prosurvival pathways, which is often found to correlate with tumor progression by directly phosphorylating and inactivating proteins, including Bad and procaspase 9 (25,26). However, reduced activity of Akt tips the balance toward apoptosis. It has been previously reported that endoplasmic reticulum stress-induced apoptosis is associated with a reduction in phospho-Akt (27,28). In addition, estrogen activates the PI3K-Akt pathway through ER α- and ER β-independent mechanisms in breast cancer (29,30). In the present study, the dephosphorylation of Akt at Ser473 was found to be involved in apoptosis induced by endoplasmic reticulum stress. In addition, simultaneous treatment with E2 at a concentration of 10^−9^ M may counteract endoplasmic reticulum stress-induced apoptosis by the Akt pathway.
